# Health Literacy of People with Intellectual Disabilities: How Meaningful Is the Social Context for a Target Group-Oriented Model of Health Literacy?

**DOI:** 10.3390/ijerph192316052

**Published:** 2022-11-30

**Authors:** Nils Sebastian Vetter, Karina Ilskens, Norbert Seidl, Änne-Dörte Latteck, Dirk Bruland

**Affiliations:** Institute for Educational and Health-Care Research in the Health Sector (InBVG), University of Applied Science Bielefeld, 33615 Bielefeld, Germany

**Keywords:** health literacy, intellectual disabilities, social context

## Abstract

Health literacy is primarily understood as an individual construct. People with intellectual disabilities still seem to be a “hidden” population in health literacy research. A target-orientated health literacy approach for this population is needed for developing precise and effective interventions that consider social context dimensions. Therefore, the following research question was answered: Which dimensions influence the health literacy of people with intellectual disabilities? A secondary data analysis containing 38 guided interviews with people with mild to moderate intellectual disabilities was conducted. The analysis followed the content analysis by Schreier (2012). Six main dimensions were inductively outlined, which are “interpersonal relationship”, “organizations and communities”, “healthcare system”, “education”, “digital interaction spaces”, “politics”, and “cultural contexts”. All these dimensions influence people with intellectual disabilities on an individual level regarding their level of health literacy. The importance of these single dimensions becomes clear, although for now, the link between all these dimensions is not yet considered. In future research, the focus should be on how to develop interventions considering social context dimensions. Furthermore, analyzing the connection between those dimensions seems promising.

## 1. Introduction

People with intellectual disabilities are a highly vulnerable group in terms of health—health literacy can empower this group [1]. Health literacy is defined as “people’s knowledge, motivation, and competences to access, understand, appraise, and apply health information in order to make judgements and decisions in everyday life concerning healthcare, disease prevention, and health promotion to maintain or improve quality of life during the life course” [2]. Latteck und Bruland 2020 [1] discussed the adequacy of the health literacy model for people with intellectual disabilities in a previous work. Based on the association of people with intellectual disabilities with reduced communicative and cognitive abilities, reading and writing skills, and self-perception, the following question is asked: “To what extent can health literacy concepts be applicable for people with intellectual disabilities?” It is assumed that people with intellectual disabilities are disadvantaged by their cognitive limitations within this understanding of health literacy [3]. Two literature reviews [4,5] confirm these assumptions. Predominantly, there is still a deficit-oriented perspective on people with intellectual disabilities. Instead, a more resource-oriented perspective should be considered. In addition, it is assumed that health literacy in people with intellectual disabilities remains on a functional level, rather than focusing on interactive or even critical health literacy, according to Nutbeam’s Health Literacy Typology [6,7]. As a result, social and communicative competencies are not addressed, although they are crucial for making health-related decisions on their own. This leads to assumptions that the level of health literacy within this group is very low [4], but so far there are no special reference sources for this statement—an adequate measuring tool is still missing [1]. Latteck and Bruland [1] analyzed three selected projects (according to the requirements of a resource-oriented approach) with the aim of defining the key factors promoting the health literacy of people with intellectual disabilities. Beside others, social context and everyday life routines are important key factors.

This definition of health literacy presented at the beginning is based on a literature review by Sørensen et al. [2], the result of which is the merging of different definitions or their main characteristics [8]. It involves the risk that health literacy is viewed as a subject-centered competency, and that it is assessed outside of the social, political, economic, and cultural contexts (decontextualization) [8]. However, there has been more attention paid to the link of social contexts and health literacy, though it is not well-conceptualized in health literacy models [9,10]. According to the Special Issue, social context is understood as interpersonal relationships, organizations, communities, health and educational systems, cultural contexts, and digital interaction spaces. For people with intellectual disabilities, Mauro et al. (2021) considered the following: “Therefore, when promoting health literacy, focus should not only be on the users, but the social components must also be taken into account, in particular, the living environment (culture) in terms of housing, work, and leisure. It is advocated that health literacy is considered as social practice, i.e., as activities that are always embedded in specific situations and contexts and whose actual form and meaning can only be understood within these contexts” [11]. This confirms that competencies and decisions (decision making) are manifested in actions, and these are linked to values, attitudes, feelings, and social relationships [8,12]. Two examples from different perspectives will illustrate these statements.

(a) People often learn how to adopt prevailing behaviors, traditions, and norms in social interactions. This learned behavior is a person’s recognition of the social context. For example, in organizations, there are certain ways to structure the workday. Adhering to this structure can help someone succeed in the company. Someone who violates certain attitudes and norms in a company may not stay in the company for long. This transfers well to the fields of housing, work, and leisure.

(b) The design of the social environment is another way to explain the social context. The social environment determines how people interact with each other; at the micro-level, how family members live and communicate with each other, and at the meso-level, how integration assistance shapes the social environment. This has a major impact on the health behaviors of individuals.

There is little that is known about the social context and people with intellectual disabilities, and research such as a review of evidence and how it is conducted for general health literacy is missing, e.g., referring to the International Classification of Functioning, Disability, and Health, the influence of environmental factors upon health conditions becomes obvious [13]. Social relationships and interactions are embraced by those environmental factors. To develop successful health literacy interventions on both the individual and collective levels, a more nuanced theory- and research-based understanding of how social contexts shape health literacy is needed [1]. For people with intellectual disabilities, there is only little knowledge about the specific link between health literacy and social context. To generate knowledge, a secondary analysis was conducted based on the results of interviews conducted in two research projects on health literacy and health behavior among people with intellectual disabilities. The guiding research question was as follows: Which dimensions influence the health literacy of people with intellectual disabilities? The focus of the analysis was on finding the dimensions of social context for the health literacy of people with intellectual disabilities and how they influence the health literacy in this target group. The results will be discussed, with the focus being for a target group-orientated health literacy interpretation. This knowledge is essential to developing adequate health-promoting measures in this high-risk group, in terms of health, and to empower them in everyday life.

## 2. Materials and Methods

Based on interviews conducted within two research projects on the topics of health literacy and applying health information (physical activity) in people with intellectual disabilities with the corresponding target group, this article aims to build a secondary analysis that analyzes the influence of the social context on the health literacy of people with intellectual disabilities. The projects are described in detail elsewhere [5,14]. Both projects were surveyed and approved by ethical review committees, which were the German Educational Research Association (No. 03/2020/DGfE) and the Ethics Committee of Bielefeld University (No. 2018-215-S).

Altogether, the projects conducted 38 guided interviews with people with mild to moderate intellectual disabilities over a period from April 2019 to September 2020. The interviewees lived alone at home, with their families, or in assisted living facilities. The interviews were conducted using problem-centered interviews [15], and the sessions were adapted to the situational and communication skills of the interviewees [4]. Interviews were recorded using an audio device and subsequently transcribed verbatim. The prerequisites for interview participation were, on the one hand, a sufficient ability to concentrate and to speak on the part of the interviewees, and on the other hand, the informed consent of the interviewees themselves, and if necessary, of a legal guardian.

A secondary analysis of the interviews was conducted with a focus on social context, following the approaches of structuring the content analysis according to Schreier (2012), using MAXQDA software [16]. At first, a coding frame was developed for defining relevant aspects in the data material. In the next step, the interviews were divided into different coding units and allocated to the coding frame. Additionally, coding frame elements were developed inductively from the interviews themselves. Finally, all the results were discussed by the researchers and placed in the overall context of health literacy.

## 3. Results

**Figure 1:**
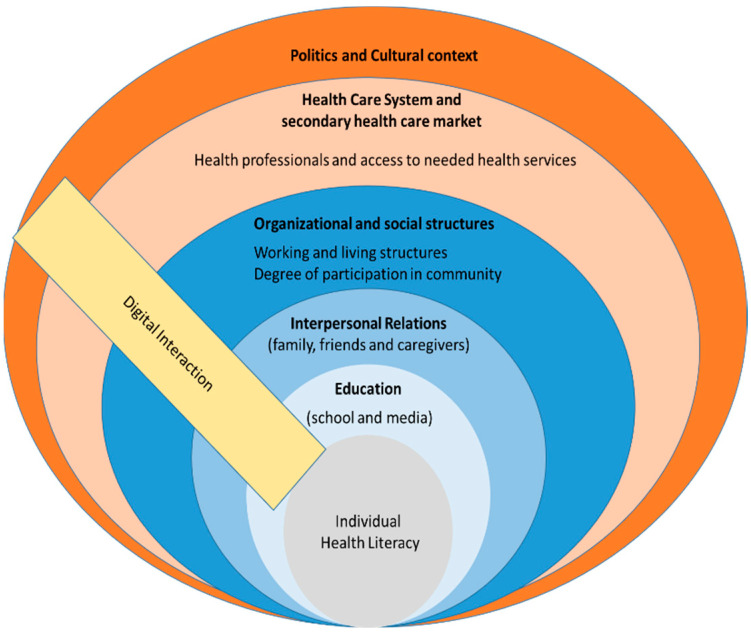
Dimensions of social context and health literacy in people with intellectual disabilities.

The health literacy of people with intellectual disabilities is significantly influenced by the social context, at different levels. The levels emerged inductively from the data material. An overview is shown in Table 1. In addition, for a better understanding, the dimensions are presented in Figure 1. The following results are structured in an inner to outer manner, beginning with the dimension of “education”.

### 3.1. Education Experience

How education is performed has an influence on health literacy in two ways. In this context, two examples from the material are worth mentioning: first, school education, and second, the transfer of knowledge through mass media, including books. The example of school education shows that the foundation for the development of health literacy can be laid at an early stage, and that schools can act as role models in this context, e.g., regarding the implementation of health-promoting behaviors such as sports activities, including an associated positive basic attitude toward health-promoting behavior.

*“B: Because running is something I’ve never said no to. I: Do you like doing it that much? B: Mhm. I did from school I always jumped out in the sandbox. We had sports at school in the summer and we had indoor sports in the winter. And in the winter also swimming badges. I: So you have always done sports. B: Always, mostly always.”*

(förges interview 1, lines 106–110)

In addition to the previously mentioned groups of people who can act as a source of health-related information for people with intellectual disabilities, the interviews also showed that media such as television, but also books, can serve as additional sources of information, and should therefore be counted among the contextual factors that influence health literacy.

*“Are you looking for information there, too? On the subject of health? (B: No.) Why not? (B: No.) No. Why don’t you do that? B: On television, there’s health. And we always watch that a lot.”*

(Geko interview 7, lines 293–295)

*“[…] my books have good advice even when stress is, you should go conflicts completely out of the way, is even in my book in it and that is actually also very interesting. There you also learn to appreciate yourself, how strong are really the body language. Because body language also says something.”*

(Geko interview 6, lines 316–321)

### 3.2. Interpersonal Relationships

Specific interpersonal relationships with people from the immediate environment, such as family members, partners, friends, or caregivers, have a high impact on the health literacy of the individual. Based on the analyzed material, this can be seen, among other things. Close caregivers are perceived as supports in maintaining health, and as an important guide to a healthy lifestyle, which strongly influences the health literacy of the person, who in turn has great trust in the caregiver in this respect.

*“B: Yes, even very much. My caregiver K (? K) also paid a lot of attention to that and I fully agree with her and I also do that without …#00:10:06#. She also says, A, we have to see what is good for you and your health, so that your body and your lungs are not overstrained, as they basically already are. So that I also take light things, half fat, half calories and so on. Vegetables and so on.”*

(Geko interview 9, lines 121–127)

In addition, the interviews also indicate that the responsible caregivers act as motivators for health-promoting behaviors.

*“Unless a supervisor comes in and motivates me a bit. But then I do it, too. (I: What do you mean, motivates you?) If a supervisor says, yes, will you come out with me? Then they try to motivate me. Because during the winter I really, really don’t like to go outside. […]. So they try to motivate me a little bit. So it works from time to time.”*

(förges interview 6, line 112)

In addition to caregivers, family members, life partners, and friends also have a significant influence on health literacy through the existing relationship of trust and emotional connection. In the interviews, it was presented, among other things, that the participation of a spouse is a significant motivating factor for active participation in sports.

*“I: But when you do exercise, you’d rather do it alone or with-. B: I’m happy when my wife is there. I: So that means you are quite a good team. B: Yes. I: So you are quite a good sports team. B: Of course. With Paderborn pants and Paderborn jersey.”*

(förges interview 2, lines 174–179)

A similar degree of importance is described for friends, who also act as a motivating factor and as pull-along persons.

*“B1: Yes, I still have a few friends who do sports with me. I’m not there alone. I: And these are the girlfriends who are here or just friends like that? B1: Yes, a friend of E’s who comes from E. I: Yes, and that’s your meeting place there, so to speak? B1: Meeting place, yes so that we do sports.”*

(förges interview 13, lines 69–73)

### 3.3. Organizational and Social Structures

In addition to the interpersonal relationships of people with intellectual disabilities and individuals within the social context, organizations and communities with which people interact have a major impact on their health literacy. Within organizations and communities, health-promoting dynamics emerge or exist that significantly influence individuals’ health literacy. From the documents evaluated, it appears that one of the roles of the employer in this context is to provide health-related information.

*“I: Was there then somehow, when you came to the workshop, again information, so that it-, B: Yes, we have-, I have there, where I arrived-, there I had to do a-, there they told me what I have to do. And then I-, so the one thought, yeah, I know what to do. Wash hands, disinfect hands, put on a mouth guard and I know that. Did I say, I have no problem with that, that I wash my hands now. Yes.”*

(Geko interview 4, lines 102–108)

In addition to the employer, residential homes and residential groups, in particular, offer opportunities for developing and strengthening health literacy through their existing structures. On the one hand, they assume a motivating role that is similar to those of persons with whom interpersonal relationships are established, and in this context, they play a key role in promoting physical activity, for example.

*“I: Yes, isn’t it? And doesn’t anyone ever come and say: “So, now get up! And now do something again!”? B: (? Yes). I: Yes, someone does? B: Yes. I: And what do you do then? For the arm? B: Arms, doing gymnastics. I: Yes. This is where you do that? B: Yes. I: At the residential facility? B: Yes. I: Ah, that’s a good idea. B: And-?”*

(förges interview 16, lines 450–461)

On the other hand, they facilitate the implementation of a healthy lifestyle through relationship-based incentives, and thus indirectly promote the health literacy of people with intellectual disabilities. According to the interview materials evaluated, fruit, for example, is provided for the residents in the residential groups.

*“B: Yes, (? I always make sure that I-). In the group now I have already eaten apples or so apple. And pears. And, what are they called, and banana I have also eaten so-, banana. I: Okay, that’s all-. That’s all fruit. B: Hold so fruit. We also did that once, so now in the group, … #00:12:34#. In the living group. … #00:12:39# I: Why is fruit important for you? Or for all people. But for you now let’s ask. B: Because of vitamins.”*

(Geko interview 10, lines 197–205)

In addition to everyday work and living structures, which as a social context influence the health literacy of people with intellectual disabilities, participation in leisure-related communities also has such an influence. For example, one interviewee described that it made him proud to already be a long-time member of the sports club, and therefore, to be honored. This attachment to the sports club could have a positive influence on the person’s health literacy.

*“B: Yes. I want one of those (? H) inside now too. I want to …#00:30:28# been. I’m 29 years in here in the club. I3: Wow. What is H? B: This is also such a, am (? currently) sports club. Have I been …#00:30:39# inside now, too. I3: Wow. 29? B: Yes. 29 years. I3: There’s a boss coming over and-. B: No no. We went to Friday, there’s also such a …#00:30:51# we were there. I …#00:30:55# were also many others honored there. I3: Great. You can be proud of that. B: Yes.”*

(förges interview 12, lines 399–407)

Finally, the degree of social participation also influences the health literacy of people with intellectual disabilities. Based on the interview material, it is clear that inclusive coexistence in communities where people with intellectual disabilities are treated with appreciation and have opportunities to participate in social life, and equal opportunities as a central factor of health literacy for people with intellectual disabilities in these communities, is clearly noticeable.

*“B: Because they also gave me such support. They support me, they accompany me. They call from time to time. During the Corona time, I also experienced that two or three community members also called and just wanted to hear how I was doing. So above all, the …#00:38:17#, the community, they give me the feeling that you belong to us, we like you, we love you, we are glad and grateful that we have you A.”*

(Geko interview 9, lines 436–441)

### 3.4. The Healthcare System—Professionals, Healthcare Services, and the Secondary Healthcare Market

In relation to the healthcare system, the material shows that the health literacy of individuals is highly dependent on the means of access to the system, with the development and promotion of literacy being primarily determined by the people working in the healthcare system, as well as the information provided. One of the interviewees reported that he received comprehensive information about his health status from healthcare professionals, which means that the staff should be considered as another significant and guiding factor in the development of health literacy.

*“Yes, they have looked, they have examined me, they have also enlightened me in this way, they have also accompanied me in this way and they have said, Mr. A, if anything is wrong, we will tell you everything, we will tell you everything that you may or may not do.”*

(Geko interview 9, lines 219–222)

However, general barriers in the system are also cited that could have a negative impact on health literacy. For example, it is confirmed that written information is usually more difficult to understand, and that a personal conversation would be preferred.

*“I: Okay. And then that one could give you more clues again. B: Yes. I: Yes, so, that is, such a sheet is not at all in order. But a conversation, well, if someone says that, then that would be super. Then that’s something great. B: Yes.”*

(Geko interview 2, lines 185–188)

Particularly regarding health professionals with whom people have contact, it is evident that trust in these individuals and their empathy have a significant influence on individual health literacy. Thus, the development and promotion of health literacy are highly dependent on individual professionals. In this context, one interviewee tells us that she has great trust in her family doctor, who listens to her and always knows what to do when she has a concern.

*“B: Yes, doctor P is general family doctor, like doctor W, but I prefer to be with doctor P, because he knows exactly, okay G has something now, now we have to look.”*

(Geko interview 6, lines 499–501)

As can be seen from the material, healthcare professionals are generally an important and reliable source of access to health information for people with intellectual disabilities. In this context, one of the interviewees affirmed that she had been sufficiently informed about her condition by hospital staff.

*“I: Did they give you good information? Did the doctors give you the-? B: Yes, they did-. Yes, they gave me good information. I: Not well? B: Yes, they did, they informed me well. I: Ah, okay. Okay. That is, as far as this heart thing is concerned, you are informed and you know what to look for. B: Yes.”*

(Geko interview 10, lines 111–117)

However, the material also shows that, despite an existing relationship of trust, there is sometimes a feeling of being ignored or not being taken seriously in discussions.

*“With my current family doctor P from T, I sometimes have the impression that he does listen to us, you can also talk to him, he’s a great guy …#00:17:13#, he only dealt with us very briefly, talked more with the staff, I don’t think that’s good.”*

(Geko interview 9, lines 203–206)

This discriminatory behavior could be seen as a factor that could have a negative effect on the development and promotion of health literacy in this target group. A comparable negative effect could result from the comprehension problems also described, e.g., speaking too quickly, which would be an indication of a lack of accessibility in communication.

*“I: And then I was in the hospital and the doctor, he was so fast, I didn’t understand that at all. Do you know this-. B: Yeah. Sometimes they talk so fast too, yeah.”*

(Geko interview 2, lines 91–92)

In addition to the factors influencing outpatient and inpatient healthcare personnel, the material studied provides evidence for the use of prescribed healthcare services (e.g., physiotherapy), which are intended to support a healthy lifestyle, and which also shape the health literacy of people with intellectual disabilities. In this regard, one interview explains that there is a certain degree of confidence that the prescribed health service, in this case, physiotherapy, will alleviate the health problem at hand (here, headaches).

*“I1: Did anything help you there? Was there anyone that helped you-, who helped you with those headaches? B: Yes, I think physiotherapy helped me a bit there.”*

(Geko interview 1, lines 195–197)

Second, prescribed health services and related professionals, such as physical therapists, act as another important source of health-related information.

*“Do you get any tips or advice from them, for example during physiotherapy, about what you can do? B: Yes. Don’t put so much strain on your back and all that. Just lifting and things like that. I2: And how did you find it? So, the tips from them? B: Yes. They are quite good.”*

(Geko interview 1, lines 221–226)

Furthermore, statements from the interview material analyzed suggest that access to secondary health market facilities, such as a gym, can improve awareness of health-promoting behaviors, which in turn promises to promote health literacy.

*“B: Well, I used to work out at the gym. There I also did something for the belly. I: Yes, and who told you that it’s good there in the gym? B: But I’m not supposed to go up there anymore, because it’s too expensive.”*

(Geko interview 3, lines 240–243)

### 3.5. Meta Level—Politics and Cultural Context

With regard to the political level, the materials evaluated show that the needs of people with disabilities continue to be given little consideration by political actors, which impairs the participation and equal rights of this target group in society and can thus make it more difficult to educate and to promote health literacy. In this context, one interviewee confirmed that a greater consideration of the needs of people with disabilities would be desirable at the local policy level, e.g., regarding the accessibility of hiking trails.

*“B: […] But these gravel roads, I hate them to death, I make more effort than anything else. I: Yes, but then-. As I said, that would be such an idea, that we-. So we can’t say ‘that’s a problem’ from Bielefeld. But for example what-. Mr. (? C) you probably know (B: Yes.), he could say, for example, as managing director, so from the city, they have to do something. Otherwise, that’s pretty disadvantageous. B: Yes, that one times for the handicapped times with, with hiking associations think along.”*

(förges interview 14, lines 57–59)

Finally, it can be deduced from the interviews that individual value, norm concepts, and intercultural differences shape health literacy at a superordinate level (meta-level). Examples that can be cited from the material in this regard relate, for example, to the perception of offers. For example, one interviewee reported that a rehabilitation sports program did not appeal to her because it was primarily attended by older people.

*“I used to do rehab sports, but I didn’t really like it because there were only older people. And I didn’t really get along with the older people.”*

(förges interview 22, line 380)

Consequently, the age-specific promotion of health literacy could prove useful. The same applies to the consideration of gender-specific characteristics. Regarding the gender-specific aspect, the statement of another interviewee makes it clear that, for example, certain physical activities (here, gardening) are perceived as being not suitable for one’s own gender.

*“I: Dancing, gardening. B: Yes, that’s not for me! I: No, gardening is not for you, is it? B: No. Only for men! I: Is only for men!”*

(förges interview 16, lines 376–380)

Finally, there are indications in the materials that, for example, language barriers in communication in the healthcare system arise due to different languages of origin, which could impair the further development and promotion of health literacy as a result of not understanding information.

*“B: Yes, because the yes-. So I have nothing against foreigners. But sometimes they speak a foreign language. And I just don’t understand them.”*

(Geko interview 10, lines 357–358)

### 3.6. Digital Spaces

As is now the case in most areas of society, digital media also influence the formation and further development of health literacy in this target group, in addition to the classic mass media (“television” and “books”). Since digital media were discussed several times in the materials examined, they are mentioned separately here regarding their influence on health literacy. Particularly in the role of an information provider, digital media (e.g., Internet and smartphone) seem to play a major role in the development of health literacy. Two interview statements indicate that the interviewees mostly use the Internet and associated search engines to obtain information.

*“I: […] where do you find out about it? B: Through the Internet […].”*

(Geko interview 6, lines 125–126)

*“I2: Yes. What do you do then if you don’t understand? B: Yeah, either ask someone or just google it or something. I2: Yeah. So, you also use Google, then, so to speak? B: What? I2: You also use Google then? B: Yes. Yeah. I2: Yeah. I do that, too. And then quite a lot of information comes up on Google, doesn’t it? B: Mhm. (agreeing) I2: How do you look up that you then find the information that is good for you? B: Yeah, if that, if that’s a foreign word, I type that in and then it tells you that, right?”*

(Geko interview 1, lines 611–623)

The smartphone, including apps and social media, also plays a central role as a provider of information, as can be seen from the interviews. For example, one interviewee explains that her smartphone—even if she cannot read—serves as an essential source of information, and thus also appears to be of great importance for health literacy.

*“I: You have a smartphone like me, a cell phone. Do you sometimes use it to find things? B: Yes, I get everything out of the cell phone. I can’t read, but I dabble everywhere. I look here and there. It took me like two days on the cell phone to get a handle on it. Yes, I have it under control.”*

(Geko interview 4, lines 298–302)

On the use of apps, another person reported using a radio station’s app when they wanted to find out about COVID-19 caseloads.

*“Yes, yes, so I, I inform myself via the app from Radio A. The case numbers are always shown there, the new ones. How many cases we have again or not. Every day.”*

(Geko interview 1, lines 61–63)

An interview with a third person also revealed that she used Facebook as an information medium in addition to other media.

*“Through the media and Facebook […].”*

(Geko interview 12, line 19)

## 4. Discussion

Individual health literacy does not automatically lead to better health outcomes. Health literacy must be seen within the contextual factors [17]. Our results support this statement. Secondary data analysis allowed us to identify and to structure different dimensions of the social context that affect health literacy among people with intellectual disabilities. In general health literacy research, this has been discussed in different places (see below) and for different target groups, e.g., Schulenkorf et al. [18]. For the first time, this has been performed for the health literacy of people with intellectual disabilities. The discussion focuses on the influence of social context and on the influence of decision making, with a focus on a target group-orientated health literacy interpretation. The various dimensions are discussed, along with examples of how health literacy can be strengthened in a target group-oriented manner. For a better understanding, the discussion is structured into the identified dimensions, beginning with the comprehensive dimension, followed by more specific ones.

### 4.1. Political and Cultural Domains

As the results show, social context has a high degree of influence, both positive and negative. It should also be pointed out that the social context contains power structures. The principle of egalitarian difference does not prevail in the social context [19]. Sociologists study people to learn more about the social context, and how and when the social context can turn from good to bad. This can involve a shift in power or balance from one side to the other. We see this in the divisive world of politics and in people trying to protect and promote their own interests. People in this social environment are often pressured to conform to the ways of the group to show the unity of the members. This is very complex, and therefore, it cannot be dealt with within this article. For example, in Germany, the fourth stage of the Federal Participation Act will be implemented in 2023. The effects can only be estimated, and they are not comparable with international developments.

### 4.2. The Healthcare System and the Secondary Healthcare Market

Our results show that how information is provided is highly important for people with intellectual disabilities. People with intellectual disabilities experience health inequalities [20]. There are two aspects to consider: the health information provided and the communication to the providers. The first aspect includes the experiences and preferences of health information, as well as support for involvement in healthcare [21]. As mentioned above, information is offered mostly in easy-to-read language, but it is assumed that information should be provided such that it fits into the individual experience and the resources. For example, many people with intellectual understanding do not prefer easy-to-read leaflets. Sometimes, they prefer them to the standard reading leaflets, but they would prefer bigger writing or more pictures. However, audio information seems to be missing in healthcare [21]. This presents opportunities for improving health information offerings. The second aspect is the support of healthcare. Little is reported from support from staff, e.g., in hospitals, talking directly to people with intellectual disabilities. There is a further opportunity for making target-orientated offerings, via special offerings according to the needs of people with intellectual disabilities, e.g., making appointments. It is mentioned that mostly, supporters from the social environment are offered. This seems ambivalent. Chinn pointed out that “interventions of them can fill in the gaps in knowledge and understanding…but also run the risk of deskilling the others in the interaction, by relieving them of the obligation to address communication breakdown directly themselves” [20].

### 4.3. Organizational and Social Structures, and Interpersonal Relationships

It is important to consider the competencies of a system in contact with the competencies and skills of users. There are elaborated concepts of organizational and social structures to health literacy. For example, the Ten Attributes of Health Literate Healthcare Organization points out important aspects for health literacy. Point five is “meets the needs of populations with a range of health literacy skills while avoiding stigmatization”. The conditions of organizations can be evaluated and improved to be more health literate and to better meet the needs of people with intellectual disabilities and their health literacy for the management of health information [22]. Little has been developed for people with intellectual disabilities. One example is the toolbox for strengthening health literacy in institutions of facilities in the areas of living and working, with the overall aim being to enable people to make appropriate decisions for their health [23].

The intrapersonal level is influenced toward the other mentioned aspects. Thus, the health literacy of individuals depends on the social context and individual health literacy. For example, the neighborhood is related to different aspects. As already mentioned, supporters take on a special significance, especially for support in everyday life and agreements in the healthcare system. Little has been developed for this special area. One example is the very comprehensive “health literacy guide to support the health of people with a cognitive impairment or intellectual disability” [24]. This guide contains 16 sections with different emphases on supporting health literacy and communication with health services. An important factor is the decision-making process mentioned.

### 4.4. Education Experience

The provision of health information is one aspect of education that is similar to the executions in the section on the healthcare system. The other aspect, school education, concerns the political domains. The education experience was hardly discussed within our team during the analysis. Finally, this domain is seen as being an intermediate step between individual health literacy and the social context. The experiences could be very different, even if they were in the same social context. To make them visible seems to be fundamental to understand why a person behaves the way he or she does, and this can lead to a greater understanding and promotion of health literacy and healthy behaviors.

### 4.5. Digital Spaces

The domain of digital spaces has a special role. This is found in each of the other domains. So far, all that can be said is that there is too little knowledge about digital health literacy; for example, making more concrete statements. Furthermore, there is a digital divide, because people with intellectual disabilities currently benefit the least from digitalization, which is understood as a social development that has influenced everyone’s everyday lives [24].

### 4.6. Merging the Domains—A Meta-Look

Overall, the mentioned domains are similar to other described models for special groups or regarding general health literacy [18,25]. Our findings demonstrate for the first time a social context with a focus on people with intellectual disabilities who are associated with reduced communicative and cognitive abilities, reading and writing skills, and self-perception. There are two aspects to mention: the measurement of health literacy and future directions for research on the social context.

A larger research cohort is needed to obtain valid and reliable statements about health literacy in a group, and quantitative methods are needed for this [1]. A tool can be used to track changes in health literacy, and it can be used as a reference for successful interventions. Currently, there is one known tool to measure health literacy in people with intellectual disabilities, which is adapted from the HLS-EU questionnaire [26]. Although the results are similar to the general population, it has to be discussed as to whether the tool is appropriate for measuring comprehensive health literacy, or whether this has to be added with other tools in order to obtain a better image of the health literacy of people with intellectual disabilities. This must take the influence of social context into account. In the research project GeKo-MmgB, case vignettes were developed and tested for different health issues orientated toward the MHL-Questionnaires of Jorm et al. [27]. The results were discussed, and the case vignettes were modified. For example, the experience with health issues should be more closely considered, and the assessment of health literacy needs to be made more accurate via a further elaboration of the assignment structures of the responses. Additional to the case vignettes, a questionnaire that is more closely related to the target group aspects and that identifies domains in our result can be adopted and be made usable for people with intellectual disabilities. One example is “The Health Literacy Questionnaire” from Osborne et al. [28].

As discussed, in every domain, there are examples from studies, guidelines, or knowledge that are otherwise raised. Currently missing is a link between all the domains. For example, there are connections described in the guidelines for health professionals working in organizations for people with intellectual disabilities to support the management of the healthcare system or to support organizational change. Social context is to be understood as a whole, and this can be a mandate for future research. For example, a hospital admission and discharge affect all essential aspects of the aforementioned domains. Social context goes beyond exploring individual areas; rather, it looks at the interplays of all the areas to examine and to create the possibility of needs-based opportunities, to empower people with intellectual disabilities in the healthcare system.

## 5. Conclusions

The promotion of health literacy in people with intellectual disabilities needs to be encouraged due to their disadvantage regarding health equities. There do exist interventions that aim at an improvement in health literacy, but right now, these only exist on an individual level. As the qualitative secondary data analysis outlined, there is more to consider than only individual aspects. Social context dimensions offer various opportunities for bringing health literacy interventions to a higher level. Especially, the important role of digital spaces shall be highlighted, as the results proved. Digital spaces can be found in each of the social context dimensions. In a society where digital information technologies are omnipresent, people with intellectual disabilities cannot be excluded from those digital spaces. The research demand was concluded, especially for three aspects: First, the continuation of developing effective health literacy interventions should be targeted. For now, mostly immethodical approaches are being undertaken to support health literacy in people with intellectual disabilities. As the development of interventions in this field is relatively new, the research needs to explore which interventions might work and which approaches seem promising. By identifying relevant social context dimensions, researchers can take the first steps for more methodical interventions. Second, these dimensions cannot be considered as being isolated. Therefore, the interdimensional dynamics should be examined in detail. By doing so, supportive dynamics can be used for precise intervention tailoring. For example, such dynamics are expected between the dimensions of “organization and communities” and “healthcare systems”. Third, future research should focus on tailoring the concept of health literacy to people with intellectual disabilities. Geukes, Bruland, and Latteck [4] mentioned this request already, and yet a specific understanding of what health literacy means to people with intellectual disabilities remains unclear. All three research tasks should be elaborated for closing the inequity gap between people with and without intellectual disabilities.

## Figures and Tables

**Table 1:** Levels of social context in people with intellectual disabilities.

Category	Description	Subcategories
**Education**	Possible education in the curriculum vitae.	▪ School ▪ Media (television/books)
**Interpersonal relationships**	Interpersonal relationships with other individuals have a major impact on an individual’s health literacy and health behaviors. In this context, a strong dependence on other individuals is often described.	▪ Caregivers ▪ Family or life partner ▪ Peers ▪ Neighborhood
**Organizational and social structures**	Within organizations and communities in which people interact, health-promoting dynamics emerge or exist that significantly affect individuals’ health literacy.	▪ Work ▪ Living ▪ Association life/clubs ▪ Community
**Healthcare system**	Individuals’ health literacy often depends on their access to and contact with the healthcare system, including the people who work there (e.g., physicians) and the information provided. At the same time, barriers in the system that have a negative impact on health literacy are cited.	▪ Healthcare workers ▪ Health services (prescribed) ▪ Secondary healthcare market
**Politics**	Policy actors rarely take into account the needs of people with disabilities, making health literacy development more difficult.	▪ Priority local policy
**Cultural contexts**	Own value and norm conceptions, as well as intercultural differences, have an effect on the development of health literacy, such as the perception of offers.	▪ Age ▪ Gender ▪ Language ▪ Education
**Digital interaction spaces**	The use of digital interaction spaces offers a significant opportunity to teach and to promote health literacy.	▪ Internet ▪ App offers ▪ Smartphone/computer/tablet ▪ Social media

## Data Availability

The data presented in this study are available upon request from the corresponding author.
